# The association of diabetes with colorectal cancer risk: the Multiethnic Cohort

**DOI:** 10.1038/sj.bjc.6605721

**Published:** 2010-06-08

**Authors:** J He, D O Stram, L N Kolonel, B E Henderson, L Le Marchand, C A Haiman

**Affiliations:** 1 Department of Preventive Medicine, Keck School of Medicine, University of Southern California/Norris Comprehensive Cancer Center, Los Angeles, CA, USA; 2 Epidemiology Program, Cancer Research Center of Hawaii, University of Hawaii, Honolulu, HI, USA

**Keywords:** diabetes, colorectal cancer, Multiethnic Cohort, smoking

## Abstract

**Background:**

Diabetics have been found to have a greater risk of colorectal cancer than non-diabetics.

**Methods:**

We examined whether this relationship differed by ethnic group, cancer site or tumour stage in a population-based prospective cohort, including 3549 incident colorectal cancer cases identified over a 13-year period (1993–2006) among 199 143 European American, African American, Native Hawaiian, Japanese American and Latino men and women in the Multiethnic Cohort.

**Results:**

Diabetics overall had a significantly greater risk of colorectal cancer than did non-diabetics (relative risk (RR)=1.19, 95% confidence interval (CI)=1.09–1.29, *P*-value (*P*)<0.001). Positive associations were observed for colon cancer, cancers of both the right and left colon, and cancers diagnosed at a localised and regional/distant stage. The association with colorectal cancer risk was significantly modified by smoking status (*P*_Interaction_=0.0044), with the RR being higher in never smokers (RR=1.32, 95% CI=1.15–1.53, *P*<0.001) than past (RR=1.19, 95% CI=1.05–1.34, *P*=0.007) and current smokers (RR=0.90, 95% CI=0.70–1.15, *P*=0.40).

**Conclusion:**

These findings provide strong support for the hypothesis that diabetes is a risk factor for colorectal cancer.

Colorectal cancer and type 2 diabetes share several risk factors including western diet, obesity and physical inactivity, and epidemiological studies have provided evidence to support a positive relationship between these two common diseases (Will *et al*, 1998; Nilsen and Vatten, 2001; Levi *et al*, 2002; Yang *et al*, 2005). One hypothesis purported to explain the association is the ‘hyperinsulinemia-hypothesis’ (McKeown-Eyssen, 1994; Giovannucci, 1995), whereby insulin resistance in type 2 diabetics leads to higher insulin levels, as well as an increased level of bioavailable IGF-I. The proliferative effects of insulin and IGF-I on the colon epithelium are believed to increase the potential for spontaneous mutations and have an important function in both the initiation and progression phases of carcinogenesis in colorectal cancer. This hypothesis is further supported by the findings that elevated circulating levels of C-peptide and IGF-I are associated with an increased risk of colorectal cancer (Kaaks *et al*, 2000; Sandhu *et al*, 2002; Ma *et al*, 2004; Renehan *et al*, 2004; Schoen *et al*, 2005; Jenab *et al*, 2007; Otani *et al*, 2007). Studies of the relationship between type 2 diabetes and colorectal cancer risk in racial/ethnic populations other than Whites have been limited; however, in general, they also support the positive association between these conditions (Jee *et al*, 2005; Seow *et al*, 2006; Vinikoor *et al*, 2009). We conducted a prospective analysis of the relationship between diabetes and colorectal cancer risk in the Multiethnic Cohort. This large prospective cohort includes five racial/ethnic populations (European Americans, African Americans, Native Hawaiians, Japanese Americans and Latinos) that represent a wide variation in the incidence of these diseases, as well as in the prevalence of known risk factors. Here, we report on the association between these end points by race, sex, cancer site (colon *vs* rectum and left colon *vs* right colon), stage (localised *vs* regional/distant) and in strata of established risk factors for colorectal cancer.

## Materials and methods

The Multiethnic Cohort includes 215 251 men and women in Hawaii and California (largely from Los Angeles County). The participants are primarily individuals of Native Hawaiian, Japanese, White, African American and Latino race/ethnicity, who entered the cohort between 1993 and 1996 by completing a detailed, self-administered questionnaire that obtained information on basic demographic variables, as well as several lifestyle factors (e.g. diet) and medical conditions (e.g. diabetes). Potential cohort members were identified primarily through the Department of Motor Vehicles drivers’ license files and additionally for African Americans, Health Care Financing Administration (Medicare) data files. Participants were between the ages of 45 and 75 years at the time of recruitment. In the cohort, incident colorectal cancer cases are identified through cohort linkage to population-based cancer Surveillance, Epidemiology and End Results registries, which cover Hawaii and Los Angeles County, as well as the rest of the state of California. For this analysis, linkage with these registries was complete through 31 December 2004, in Hawaii and 31 December 2006 in California. Over this period, 1921 male and 1628 female cases of colorectal cancer were identified. Deaths within the cohort are determined from linkages to the death certificate files in Hawaii and California, supplemented with linkages to the National Death Index. Diabetes status is defined based on self-report to a question on the baseline questionnaire asking whether a doctor had ever told the respondent that he/she had diabetes (high blood sugar). The question did not differentiate between type 1 and type 2 diabetes mellitus and thus we expect a small fraction (<10%) of the self-reported diabetes cases to have type 1 diabetes and be potentially misclassified. Year of diabetes diagnosis (before 1994, 1994, 1995, 1996, 1997 or 1998) was defined using a second questionnaire in 2001, and 90.3% (79 178 men and 100 663 women) of the subjects who returned the first questionnaire also returned the second questionnaire.

In the analysis, we excluded one male subject missing information for diabetes status on the baseline questionnaire. The prospective analysis of the association between diabetes status and colorectal cancer incidence in this study includes 89 478 men and 109 664 women in the Multiethnic Cohort from the five main ethnic groups.

The study protocol was approved by the institutional review boards at the University of Southern California and the University of Hawaii.

### Statistical analysis

Cox regression was used to estimate hazard ratios (reported as relative risks (RRs)) for the effect of diabetes on colorectal cancer incidence. Models were stratified by age at entry of the cohort, and were minimally adjusted for ethnicity and sex, or further adjusted for the following risk factors as potential confounders: body mass index (BMI) (<23, ⩾23–25, ⩾25–30, ⩾30–35, ⩾35 kg m^−2^ and missing), smoking status (never, past, current and missing), educational level (⩽12 years, some college or vocational, college graduate and missing), alcohol intake (never, <12, 12–24 and ⩾24 g per day and missing), non-steroidal anti-inflammatory drugs (NSAIDs) use (yes, no and missing), saturated fat intake (categorised by quartiles of the distribution of percent of calories from saturated fat; ⩽7.0%, >7.0–8.8%, >8.8–10.6%, >10.6% and missing), non-saturated fat intake (categorised by quartiles of the distribution of percent of calories from non-saturated fat; ⩽17.8%, >17.8–21.2%, >21.2–24.5%, >24.5% and missing), dietary fibre intake (categorised by quartiles of the distribution of dietary fibre intake; ⩽8.7, >8.7–11.3, >11.3–14.5, >14.5 g kcal^−1^ and missing), total calories (categorised by quartiles of the distribution of calories intake per day; ⩽1417.8, >1417.8–1918.9, >1918.9–2608.9, >2608.9 kcal per day and missing), vigorous activity (never, ⩽0.21, >0.21–0.71, >0.71 hours per day and missing), family history of colorectal cancer (yes and no), menopausal status and hormone replacement therapy (HRT) use (premenopausal, postmenopausal never HRT users, postmenopausal past HRT users, postmenopausal current HRT users, and those missing information on menopause status or HRT use) among women only. For each covariate, an indicator variable was used for those missing data.

RRs were estimated separately for colon and rectal cancer, by location in the colon (left *vs* right; 31 men and 42 women were missing information for this variable and were excluded from this analysis) and by stage (localised *vs* regional/distant; 190 men and 213 women were missing information for this variable and were excluded from this analysis). We also examined whether the association may be modified by known risk factors (at baseline): age (<60, 60–69 and ⩾70 years), BMI (<25 and ⩾25 kg m^−2^; 881 men and 2704 women were missing information for BMI and were further excluded from this analysis), smoking status (never, past and current; 1034 men and 2145 women were missing information for smoking status and were further excluded from this analysis) and NSAIDs use (yes and no; 2984 men and 5701 women were missing information for NSAIDs use and were further excluded from this analysis).

The risk of colorectal cancer in diabetics has been reported to be highest 10–15 years after diabetes diagnosis, with risk declining in later years perhaps as a result of hypoinsulinemia (La Vecchia *et al*, 1997; Le Marchand *et al*, 1997; Hu *et al*, 1999). In an attempt to assess the association between time since diabetes diagnosis and colorectal cancer risk, we performed analysis comparing colorectal cancer risk in long-term and short-term diabetics. Subjects who reported a diabetes diagnosis before 1994 were considered to have a long duration of diabetes, and those who reported a diabetes diagnosis in or after 1994 were considered to have a short duration of diabetes. In this analysis, we began the follow-up at the time the second questionnaire was returned (2001). In all, 5344 diabetic men and 5842 diabetic women were missing information for diagnosis year of diabetes or did not return the second questionnaire, or had colorectal cancer before the return date of the second questionnaire and thus were excluded from this analysis. After exclusion, 9716 diabetic men and 10 429 diabetic women were included in this analysis.

## Results

The mean age of the men (*n*=89 478) at baseline of the cohort was 60.2 years (s.d., 8.9), which ranged from 57.2 years for Native Hawaiians to 62.5 years for African Americans. The prevalence of diabetes varied widely across populations, from 9.5% in European Americans to 21.6% in Latinos. Diabetic men were slightly older than non-diabetic men for each racial/ethnic group, ranging from 62.7 years (*vs* 59.2 in non-diabetics) in European Americans to 61.9 years (*vs* 60.3 in non-diabetics) in Latinos. Among men, the age-standardised incidence rate of colorectal cancer was higher in diabetics than non-diabetics among European Americans, African Americans, Japanese Americans and Latinos, but lower in Native Hawaiians. In each population, diabetic men were more likely to be overweight and less physically active than men without diabetes (Table 1).

The mean age of the women (*n*=109 664) was 59.7 years (s.d., 8.9), with Native Hawaiians being the youngest (mean, 56.7 years) and Japanese and African Americans being the oldest (mean, 61.4 years; Table 1). Among women, the prevalence rate of diabetes was lowest in European Americans (7.8%) and highest in African Americans (20.0%). Likewise, the mean age of diabetic women at baseline was greater than non-diabetic women. Age-standardised rates of colorectal cancer in women were greater in diabetics than non-diabetics for all populations except Native Hawaiians. Among women, obesity and physical inactivity were consistently associated with diabetes across populations (Table 1).

The associations noted by race and sex were not materially modified after adjustment for potential risk factors for colorectal cancer; none of the risk factors served as strong independent confounders of the association between colorectal cancer risk and diabetes (Table 2). In multivariate analyses, the RR of colorectal cancer in diabetics was 19% higher compared with non-diabetics (RR=1.19, 95% confidence interval (CI)=1.09–1.29, *P*<0.001; Table 2). The RR was greater and more significant in women (RR=1.28, 95% CI=1.12–1.46, *P*<0.001) than in men (RR=1.12, 95% CI=0.99–1.26, *P*=0.063; *P*_Interaction_=0.071). A positive association between diabetes and colorectal cancer risk was noted in all ethnic groups except among Native Hawaiians, with the RR estimates ranging from 1.16 in European Americans (95% CI=0.91–1.48, *P*=0.24) and African Americans (95% CI=0.97–1.38, *P*=0.097) to 1.27 in Japanese Americans (95% CI=1.09–1.47, *P*=0.002). Risk was non-significantly lower in Native Hawaiians (RR=0.89, 95% CI=0.62–1.27, *P*=0.52); however, a test of heterogeneity suggested no significant difference in the risk estimates across ethnic groups (*P*_Interaction_=0.43) (Table 2).

In analyses stratified by cancer site (colon *vs* rectum) (Table 2), risk of colon cancer was elevated in diabetics (RR=1.20, 95% CI=1.09–1.32, *P*<0.0001) as was rectal cancer risk (RR=1.15, 95% CI=0.96–1.36, *P*=0.12). Test of heterogeneity by ethnicity was not significant for colon cancer (*P*_Interaction_=0.30) or for rectal cancer (*P*_Interaction_=0.18). However, for rectal cancer, a particularly strong association between cancer risk and diabetes was observed only in Latinos (RR=1.55, 95% CI=1.13–2.14, *P*=0.007) (Table 2), with the association noted in Latino men (RR=1.51, 95% CI=1.00–2.27, *P*=0.050) and women (RR=1.61, 95% CI=0.97–2.69, *P*=0.067) (Supplementary Tables 1 and 2). The risk of cancer associated with diabetes was elevated and similar in magnitude for cancer in the right colon (RR=1.23, 95% CI=1.08–1.41, *P*=0.002) and in the left colon (RR=1.18, 95% CI=1.01–1.38, *P*=0.040). For both locations, tests of heterogeneity suggested no difference across ethnic groups (Table 2).

In analyses by stage (Table 2), there were significant increases in risk for both regional/distant cancer and localised cancer (RR=1.21, 95% CI=1.06–1.39, *P*=0.005 for localised cancer; RR=1.14, 95% CI=1.01–1.30, *P*=0.036 for regional/distant cancer). No significant heterogeneity across ethnic groups was observed by stage. For regional/distant colorectal cancer, the RR was similar in men (RR=1.15, 95% CI=0.97–1.36, *P*=0.10) and women (RR=1.14, 95% CI=0.94–1.37, *P*=0.19); whereas for localised cancer, there was no significant increase in risk observed in diabetic men (RR=1.06, 95% CI=0.88–1.27, *P*=0.55), but the risk was significantly increased in diabetic women (RR=1.44, 95% CI=1.18–1.76, *P*<0.001; *P*_Interaction_=0.0098) (Supplementary Tables 1 and 2).

We observed no significant difference in the association between diabetes status and colorectal cancer risk by BMI (⩾25 kg m^−2^: RR=1.21, 95% CI=1.09–1.34; <25 kg m^−2^: RR=1.23, 95% CI=1.05–1.45, *P*_Interaction_=0.14; Table 3). Nor were significant differences noted in the association when stratified by age, NSAIDs use, or HRT use (among women). However, we found that the association between diabetes status and colorectal cancer risk differed significantly by smoking status (*P*_Interaction_=0.0044). The association was strongest in never smokers, with a RR of 1.32 (95% CI=1.15–1.53, *P*<0.001); in past smokers, the RR was 1.19 (95% CI=1.05–1.34, *P*=0.007); no positive association was observed in current smokers (RR=0.90, 95% CI=0.70–1.15, *P*=0.40). This pattern was generally consistent in both sexes and in all colorectal cancer subgroups (Supplementary Table 3), and a significant interaction between tobacco use and diabetes status was observed in men, and for regional/distant colorectal cancer and rectal cancer. We observed no heterogeneity by ethnicity within a defined strata for any risk factor (e.g. current smokers; Table 3).

We also investigated the relationship between time since diagnosis with diabetes and colorectal cancer risk. Compared with diabetics diagnosed in 1994 or later, those with an earlier diagnosis were at non-significantly lower risk of colorectal cancer risk (RR_long-duration *vs* short-duration_=0.89, 95% CI=0.69–1.14, *P*=0.35).

## Discussion

In the prospective analysis of five racial/ethnic populations in the Multiethnic Cohort, we found a highly statistically significant association between diabetes status and colorectal cancer incidence, with diabetics having 19% greater risk of developing colorectal cancer than non-diabetics after adjusting for known risk factors. The risk associated with diabetes was slightly greater in women (RR=1.28) than in men (RR=1.12) (*P*_Interaction_=0.071), and the positive association was observed in all populations except Native Hawaiians, which was the smallest group.

Only a small number of studies have investigated the relationship between diabetes and colorectal cancer risk in non-European populations and even fewer have closely examined the association by colorectal cancer site, stage and other known risk factors for colorectal cancer across multiple ethnic groups. Our findings in Japanese Americans suggested that risk of colorectal cancer was elevated in diabetics in both men and women (RR=1.15, 95% CI=0.95–1.39 in men and RR=1.49, 95% CI=1.18–1.91 in women), which is consistent with earlier reports in Koreans and Singapore Chinese (Jee *et al*, 2005; Seow *et al*, 2006). In contrast to earlier report of no association with colon or rectal cancer among African Americans (Vinikoor *et al*, 2009), we found that diabetes was significantly associated with colon cancer risk in African Americans (RR=1.24, 95% CI=1.02–1.49), but not with rectal cancer risk (RR=0.85, 95% CI=0.54–1.32). We also found no evidence of an association of diabetes and colorectal cancer risk in Native Hawaiians, which has not been reported before.

In analyses by cancer site, risk was significantly increased for colon cancer, cancer of the right colon and left colon. Non-significant overall positive associations were observed for rectal cancer. For cancer of the rectum, although the test for interaction suggested no heterogeneity in the association across ethnic groups, we did notice that the risk or colorectal cancer association with diabetes was significantly elevated in Latinos (RR=1.51 in men and RR=1.61 in women), but not in any of the other ethnic groups. In analyses by stage, significant increases in risks were noted for both regional/distant and localised disease. Risks within tumour subgroups were generally consistent across ethnic groups. The relationship between diabetes status and colorectal cancer was significantly modified by smoking status. In contrast to an earlier report of greater colorectal cancer risk in current and former smokers than never smokers among diabetics (Limburg *et al*, 2006; Gibbs *et al*, 2007), the association in our study was stronger in never smokers (RR=1.32) than in past smokers (RR=1.19), whereas no positive association was observed in current smokers (RR=0.90). With smoking being a risk factor for colorectal cancer, it is possible that the association between diabetes and colorectal cancer risk may be more apparent in those at lower risk. Additional studies will be needed to better understand this observation.

In this study, diabetes status was based on self-report, which may have led to some misclassification of diabetics as non-diabetics. This underreporting may have resulted in an underestimate of the magnitude of the association between diabetes and colorectal cancer risk. However, earlier studies have shown that self-reported responses for many common chronic diseases such as diabetes are reliable when compared with medical records (Midthjell *et al*, 1992; Okura *et al*, 2004; Walitt *et al*, 2008). We could not differentiate between cases with type 1 and type 2 diabetes; however, we expect this misclassification to be minimal as type 1 diabetes is uncommon. Another limitation of our study is that the analysis did not account for incident cases of diabetes over the follow-up period. Incident cases of diabetes in the non-diabetic group during the follow-up would make the two groups more similar and therefore attenuate the true association between diabetes and colorectal cancer risk.

With ⩽13 years of follow-up, we were unable to address the effects of long-standing diabetes on colorectal cancer risk. However, we observed that diabetics diagnosed before 1994 were at lower risk of having colorectal cancer compared with those who were diagnosed with diabetes in 1994 or later (RR=0.89). These findings support the results from earlier studies (La Vecchia *et al*, 1997; Le Marchand *et al*, 1997; Hu *et al*, 1999) and the hyperinsulinemia-hypothesis, according to which risk of colorectal cancer should be lower in later years of diabetes as a result of hypoinsulinemia.

In this large, multiethnic prospective study, we observed consistent positive associations between diabetes and colorectal cancer risk in African Americans, Latinos, Japanese and European Americans. The lack of positive association between diabetes and colorectal cancer risk in Native Hawaiians is consistent with the relatively low rate of colorectal cancer in this group despite a high rate of diabetes (Grandinetti *et al*, 2007). Additional follow-up and larger studies will be needed to confirm the apparent inverse association in Native Hawaiians. These findings provide strong support for the hypothesis that diabetes is a risk factor for colorectal cancer.

## Figures and Tables

**Table 1:** Baseline characteristics of the participants, by ethnicity and by diabetes status

	**European Americans**		**African Americans**		**Native Hawaiians**		**Japanese Americans**		**Latinos**	
Characteristic	**Diabetics**	**Non-diabetics**	**Diabetics**	**Non-diabetics**	**Diabetics**	**Non-diabetics**	**Diabetics**	**Non-diabetics**	**Diabetics**	**Non-diabetics**
	Men									
No. of subjects (%)	2157 (9.5)	20 471 (90.5)	2573 (20.6)	9944 (79.6)	1341 (21.5)	4897 (78.5)	4297 (16.3)	22 059 (83.7)	4692 (21.6)	17 047 (78.4)
Mean age, years (s.d.)	62.7 (8.4)	59.2 (9.1)	64.0 (8.1)	62.1 (9.0)	58.8 (8.2)	56.8 (8.7)	63.4 (8.5)	61.2 (9.3)	61.9 (7.2)	60.3 (7.9)
No. of colorectal cancer cases	41	318	67	256	24	93	134	578	105	305
Colorectal cancer incidence rates^a^	126.1	106.9	160.2	139.6	137.7	155.7	164.4	164.3	125.1	99.1
Family history of colorectal cancer, %^b^	7.6	8	7.4	6.5	6.1	6.5	9.9	9.8	3.8	3.7
										
*Body mass index (kg m*^−*2*^), %^c^										
<23	7.4	18.2	7.9	14.7	4.9	11.4	15.9	25.9	7.4	11.1
⩾23–25	11.1	21.8	10.6	17.4	7.6	14.2	20.3	26.8	12.3	17
⩾25–30	43.3	45.6	43.3	46.4	38.3	45.9	46.2	40.8	47.8	53.9
⩾30–35	24.5	11.4	24.6	14.5	27.6	19.5	13.5	5.5	24	14
⩾35	13.1	2.7	9.9	3.9	19.9	8	3.9	0.8	7.7	3.2
Missing	0.5	0.4	3.3	3.1	1.7	1	0.2	0.2	0.8	0.9
										
*Educational level*, %^c^										
⩽12 years	32.6	23	41.1	40.4	60.5	53.4	36.7	35	64.9	63.4
Some college or vocational	30.6	28.5	36.7	35.8	25.8	28	33.2	30.1	23	22.8
College graduate	36.3	47.7	20.7	22.2	12.8	17.4	29.5	34.3	10.4	12
Missing	0.5	0.8	1.1	1.6	0.9	1.2	0.7	0.6	1.7	1.8
										
*Physical activity (hours per day)*, %^c^										
Never	38.9	27.9	41.9	34.5	29.2	22.3	39.2	33	36.3	29
⩽0.21	16.3	14.4	18.1	15.1	14.3	13.2	18.9	18.1	14.4	14.2
>0.21–0.71	20.9	23.4	17.3	21.3	24	25.6	21.8	23.4	18.6	20.8
>0.71	20.3	31.5	17.4	24.2	28.3	35.6	17.4	23	25.2	30.8
Missing	3.6	2.8	5.2	4.9	3.7	3.4	2.7	2.5	5.5	5.1
										
*NSAIDs use*, %^c^										
No	42.3	49.8	46.8	51.2	55.2	61.1	58.4	67.9	53.3	55.7
Yes	55.1	48	48.6	44.2	41.6	35.4	39.4	30.2	41.3	38.8
Missing	2.7	2.1	4.6	4.6	3.2	3.5	2.2	1.8	5.4	5.5
										
	Women									
No. of subjects (%)	2065 (7.8)	24 355 (92.2)	4372 (20.0)	17 481 (80.0)	1548 (19.0)	6587 (81.0)	3629 (12.2)	26 024 (87.8)	4657 (19.7)	18 946 (80.3)
Mean age, years (s.d.)	62.1 (8.4)	59.2 (9.0)	63.0 (8.5)	61.0 (9.2)	58.1 (8.5)	56.4 (8.8)	63.2 (8.6)	61.2 (9.0)	61.4 (7.3)	59.3 (7.8)
No. of colorectal cancer cases	38	267	111	357	16	72	90	395	68	214
Colorectal cancer incidence rates^a^	107.5	70.1	138.6	110.9	69.3	80	179.2	95.8	81.6	59.7
Family history of colorectal cancer, %^b^	8.8	9.2	8.7	8.4	7.3	7.2	11.1	11.1	4.5	5.1
										
*Body mass index (kg m*^−*2*^), %^c^										
<23	11.1	36.4	5.3	13.8	7.8	20.4	25.8	50.5	6.9	17.2
⩾23–25	9.6	19.5	6.1	14.3	8.1	16.6	16.8	20.7	10.2	18.2
⩾25–30	29.5	28	28.4	36.1	30.1	32.7	36.4	21.8	35	39.5
⩾30–35	24.4	10.2	26.7	20.1	24.9	17.3	14.9	4.3	28.1	16.5
⩾35	22.9	4.9	27	11.7	24.1	10.3	4.4	1	17	6.8
Missing	2.5	1.0	5.8	4.1	5	2.8	1.7	1.8	2.7	1.8
										
*Educational level*, %^c^										
⩽12 years	43.6	30.2	46.4	38.5	65.2	58.7	41.8	39.1	75.4	70.7
Some college or vocational	30.1	33.2	33.8	37.2	22.6	26.1	31.2	29.6	16.6	18.9
College graduate	25.4	35.7	17.6	22.8	11.3	14.1	26.3	30.5	6.1	8.2
Missing	0.9	0.9	1.5	1.5	0.9	1.1	0.7	0.8	1.9	2.1
										
*Physical activity (hours per day)*, %^c^										
Never	57.9	48.5	60.3	56.1	47.7	43.7	61.7	59.5	58.1	53.2
⩽0.21	15.6	15.5	15.3	17	17.9	16.8	16.2	15.8	13.5	15.3
>0.21–0.71	12	16.9	9.7	11.5	15.8	19.4	12.2	13.5	10.4	12.3
>0.71	9.3	14.6	5.3	7.3	11.5	15.1	6.3	7.5	7.1	9.6
Missing	5.1	4.5	9.3	8.1	6.4	5	3.6	3.7	10.8	9.6
										
*NSAIDs use*, %^c^										
No	49.6	54.9	45.1	50.2	58.6	63.2	68.9	75	49.4	53.7
Yes	46.7	41.9	46.9	43	34.5	31.5	27.7	22.1	41.5	38.5
Missing	3.7	3.2	8	6.8	6.9	5.4	3.4	2.9	9.1	7.8
										
*HRT use*, %^d^										
Never	48.8	42.3	59.4	57.7	63.6	55.9	51.4	49.2	57.9	54.8
Past	20.9	17.6	20.1	18.7	15.1	16.1	14.7	12.5	17.7	16.6
Current	26.2	37.1	13.2	17.7	15.4	23.5	30.3	34.8	15.8	19.9
Missing	4.1	3	7.3	6	5.9	4.5	3.6	3.4	8.6	8.8

Abbreviations: HRT=hormone replacement therapy; NSAIDs=non-steroidal anti-inflammatory drugs.

a Age standardised (5-year age groups) to 2000 US standard population with age 40 years old or above, per 100 000 person-years.

b Age standardised (5-year age groups) to the total population included in the study, subjects who were missing information for family history of colorectal cancer were included as with no family history.

c Age standardised (5-year age groups) to the total population included in the study.

d Age standardised (5-year age groups) to the total women included in the study.

**Table 2:** Age-adjusted^a^ and multivariate^b^ relative risks of colorectal cancer associated with diabetes status according to ethnic or racial group, site and stage of disease in the Multiethnic Cohort

	**European Americans**			**African Americans**			**Native Hawaiians**			**Japanese Americans**			**Latinos**			**All**		
		**RR (95% CI)**			**RR (95% CI)**			**RR (95% CI)**			**RR (95% CI)**			**RR (95% CI)**			**RR (95% CI)**	
	**Cases**	**Age-adj**	**Multi**	**Cases**	**Age-adj**	**Multi**	**Cases**	**Age-adj**	**Multi**	**Cases**	**Age-adj**	**Multi**	**Cases**	**Age-adj**	**Multi**	**Cases**	**Age-adj**	**Multi**
*All*	664	1.26 (0.99–1.59)	1.16 (0.91–1.48)	791	1.16 (0.98–1.37)	1.16 (0.97–1.38)	205	0.91 (0.64–1.29)	0.89 (0.62–1.27)	1197	1.29 (1.12–1.50)	1.27 (1.09–1.47)	692	1.24 (1.05–1.48)	1.21 (1.02–1.46)	3549	1.22 (1.12–1.33)	1.19 (1.09–1.29)
*P*-value		0.058	0.24		0.084	0.097		0.59	0.52		0.001	0.002		0.013	0.030		<0.001	<0.001
*P*_Int_^c^																	0.43	0.43
																		
*Men*	359	1.07 (0.77–1.48)	0.96 (0.69–1.35)	323	1.01 (0.77–1.33)	1.02 (0.77–1.34)	117	0.95 (0.60–1.49)	0.97 (0.60–1.55)	712	1.15 (0.95–1.38)	1.15 (0.95–1.39)	410	1.25 (1.00–1.56)	1.31 (1.04–1.64)	1921	1.12 (1.00–1.26)	1.12 (0.99–1.26)
*P*-value		0.69	0.83		0.93	0.91		0.82	0.90		0.15	0.16		0.048	0.023		0.048	0.063
*P*_Int_^c^																	0.66	0.65
																		
*Women*	305	1.52 (1.08–2.13)	1.48 (1.03–2.12)	468	1.27 (1.02–1.57)	1.27 (1.02–1.58)	88	0.89 (0.52–1.54)	0.83 (0.47–1.46)	485	1.56 (1.24–1.97)	1.50 (1.18–1.91)	282	1.25 (0.95–1.64)	1.09 (0.82–1.45)	1628	1.34 (1.19–1.52)	1.28 (1.12–1.46)
*P*-value		0.017	0.033		0.030	0.036		0.69	0.51		<0.001	0.001		0.11	0.54		<0.001	<0.001
*P*_Int_^c^																	0.32	0.32
																		
*Colon*	501	1.33 (1.02–1.73)	1.20 (0.92–1.59)	642	1.25 (1.04–1.50)	1.24 (1.02–1.49)	144	0.87 (0.57–1.32)	0.84 (0.54–1.30)	847	1.35 (1.14–1.60)	1.34 (1.13–1.60)	494	1.13 (0.91–1.39)	1.10 (0.88–1.36)	2628	1.24 (1.12–1.36)	1.20 (1.09–1.32)
*P*-value		0.035	0.18		0.018	0.028		0.50	0.43		0.001	0.001		0.26	0.40		<0.001	<0.001
*P*_Int_^c^																	0.30	0.30
																		
*Right colon*	307	1.40 (1.00–1.95)	1.32 (0.93–1.87)	397	1.18 (0.93–1.49)	1.17 (0.92–1.49)	68	1.06 (0.60–1.88)	1.17 (0.64–2.14)	423	1.47 (1.16–1.86)	1.46 (1.14–1.85)	269	1.08 (0.81–1.43)	1.07 (0.79–1.43)	1464	1.26 (1.11–1.43)	1.23 (1.08–1.41)
*P*-value		0.050	0.12		0.17	0.21		0.84	0.60		0.0010	0.002		0.61	0.67		<0.001	0.002
*P*_Int_^c^																	0.45	0.47
																		
*Left colon*	182	1.34 (0.86–2.07)	1.14 (0.72–1.80)	223	1.39 (1.03–1.88)	1.38 (1.01–1.89)	71	0.68 (0.36–1.31)	0.61 (0.31–1.18)	406	1.26 (0.98–1.63)	1.27 (0.98–1.64)	209	1.15 (0.84–1.59)	1.10 (0.79–1.53)	1091	1.23 (1.06–1.43)	1.18 (1.01–1.38)
*P*-value		0.20	0.58		0.032	0.044		0.25	0.14		0.069	0.077		0.39	0.59		0.008	0.040
*P*_Int_^c^																	0.36	0.34
																		
*Rectum*	163	1.03 (0.61–1.73)	1.00 (0.59–1.71)	149	0.81 (0.53–1.25)	0.85 (0.54–1.32)	61	1.02 (0.55–1.88)	1.01 (0.53–1.91)	350	1.16 (0.88–1.54)	1.10 (0.83–1.47)	198	1.57 (1.15–2.13)	1.55 (1.13–2.14)	921	1.17 (0.99–1.38)	1.15 (0.96–1.36)
*P*-value		0.91	1.00		0.34	0.47		0.96	0.99		0.29	0.51		0.004	0.007		0.071	0.12
*P*_Int_^c^																	0.15	0.18
																		
*Localised*	277	1.39 (0.97–1.97)	1.22 (0.84–1.75)	278	1.31 (1.00–1.72)	1.32 (1.00–1.76)	102	0.97 (0.60–1.57)	0.95 (0.57–1.57)	551	1.33 (1.07–1.65)	1.26 (1.01–1.56)	252	1.18 (0.88–1.57)	1.19 (0.88–1.61)	1460	1.27 (1.11–1.44)	1.21 (1.06–1.39)
*P*-value		0.070	0.30		0.053	0.052		0.89	0.83		0.009	0.040		0.27	0.25		<0.001	0.005
*P*_Int_^c^																	0.73	0.70
																		
*Regional*	317	1.15 (0.81–1.63)	1.07 (0.74–1.54)	377	0.95 (0.74–1.23)	0.95 (0.73–1.24)	97	0.92 (0.55–1.51)	0.87 (0.52–1.47)	579	1.25 (1.01–1.54)	1.24 (1.00–1.54)	316	1.39 (1.09–1.78)	1.33 (1.03–1.72)	1686	1.17 (1.03–1.32)	1.14(1.01–1.30)
*P*-value		0.45	0.72		0.71	0.73		0.73	0.61		0.042	0.052		0.009	0.029		0.013	0.036
*P*_Int_^c^																	0.19	0.21

Abbreviations: BMI=body mass index; CI=confidence interval; NSAIDs=non-steroidal anti-inflammatory drugs; RR=relative risk.

a Adjusted for sex (except in models for men/women), race (except in models for each ethnic group), and and stratified on age at baseline questionnaire.

b Further adjusted for BMI, smoking, NSAIDs use, education, alcohol intake, saturated fat intake, unsaturated fat intake, dietary fibre intake, physical activity, family history of colorectal cancer.

c *P*-value for interaction between ethnic group and diabetes.

**Table 3:** Multivariate relative risks^a^ of colorectal cancer associated with diabetes status according to risk factors in the Multiethnic Cohort

	**European Americans**		**African Americans**		**Native Hawaiians**		**Japanese Americans**		**Latinos**		**All**			
	**Cases**	**RR (95% CI)**	**Cases**	**RR (95% CI)**	**Cases**	**RR (95% CI)**	**Cases**	**RR (95% CI)**	**Cases**	**RR (95% CI)**	**Cases**	**RR (95% CI)**	***P*_Int_** b	***P*_Int_** c
*BMI (kg m*^−*2*^)														
<25	266	0.86 (0.46–1.58)	205	1.63 (1.11–2.38)	46	1.67 (0.68–4.13)	695	1.16 (0.92–1.45)	184	1.36 (0.92–2.01)	1396	1.23 (1.05–1.45)	0.48	0.14
*P*-value		0.62		0.012		0.27		0.21		0.12		0.012		
⩾25	389	1.31 (1.00–1.71)	557	1.08 (0.88–1.31)	156	0.86 (0.58–1.27)	493	1.43 (1.17–1.76)	493	1.19 (0.97–1.46)	2088	1.21 (1.09–1.34)	0.13	
*P*-value		0.047		0.46		0.45		<0.001		0.091		<0.001		
														
*Age (years)*														
<60	167	1.22 (0.70–2.14)	227	0.93 (0.65–1.33)	89	1.08 (0.65–1.82)	290	1.23 (0.89–1.72)	199	1.00 (0.69–1.45)	972	1.08 (0.91–1.29)	0.63	0.059
*P*-value		0.48		0.69		0.76		0.21		1.00		0.39		
60–69	281	1.37 (0.98–1.92)	299	1.31 (1.00–1.71)	89	0.82 (0.47–1.42)	507	1.43 (1.14–1.78)	363	1.37 (1.08–1.74)	1539	1.33 (1.17–1.50)	0.19	
*P*-value		0.066		0.053		0.48		0.002		0.009		<0.001		
⩾70	216	0.82 (0.51–1.32)	265	1.17 (0.87–1.57)	27	0.61 (1.17–2.19)	400	1.06 (0.81–1.38)	130	1.15 (0.76–1.74)	1038	1.05 (0.89–1.23)	0.38	
*P*-value		0.42		0.30		0.44		0.67		0.50		0.58		
														
*Smoking*														
Never	211	1.03 (0.65–1.63)	287	1.35 (1.02–1.79)	60	1.07 (0.56–2.05)	484	1.54 (1.22–1.95)	259	1.25 (0.93–1.68)	1301	1.32 (1.15–1.53)	0.55	0.0044
*P*-value		0.90		0.038		0.83		<0.001		0.14		<0.001		
Past	325	1.38 (1.00–1.90)	347	1.04 (0.81–1.35)	99	0.75 (0.45–1.26)	528	1.30 (1.05–1.61)	327	1.21 (0.94–1.56)	1626	1.19 (1.05–1.34)	0.18	
*P*-value		0.047		0.74		0.28		0.016		0.14		0.007		
Current	121	0.68 (0.31–1.49)	151	1.14 (0.74–1.76)	43	1.18 (0.49–2.84)	175	0.54 (0.31–0.94)	93	1.18 (0.71–1.98)	583	0.90 (0.70–1.15)	0.25	
*P*-value		0.33		0.54		0.71		0.029		0.52		0.40		
														
*NSAIDs*														
No	349	1.05 (0.73–1.50)	387	1.06 (0.81–1.37)	123	0.83 (0.51–1.35)	837	1.35 (1.13–1.63)	368	1.19 (0.92–1.52)	2064	1.17 (1.04–1.32)	0.24	0.76
*P*-value		0.81		0.69		0.45		0.001		0.18		0.008		
Yes	291	1.25 (0.88–1.78)	352	1.27 (0.99–1.63)	75	0.96 (0.55–1.68)	324	1.09 (0.83–1.43)	283	1.27 (0.97–1.68)	1325	1.20 (1.04–1.37)	0.94	
*P*-value		0.21		0.058		0.88		0.54		0.085		0.10		
														
*Menopausal status and HRT use* d														
Pre	12	8.74 (1.30–58.70)	21	0.47 (0.088–2.55)	13	0.79 (0.15–4.26)	29	4.83 (1.95–11.95)	13	2.83 (0.40–19.91)	88	2.04 (1.17–3.57)	0.040	0.080
*P*-value		0.026		0.38		0.79		0.001		0.30		0.012		
Post never	124	1.34 (0.79–2.27)	209	1.60 (1.17–2.19)	40	0.57 (0.24–1.37)	212	1.42 (0.99–2.03)	136	1.01 (0.67–1.52)	721	1.30 (1.08–1.57)	0.26	
*P*-value		0.28		0.003		0.21		0.059		0.96		0.005		
Post past	74	1.42 (0.67–2.97)	116	1.00 (0.62–1.62)	16	1.65 (0.28–9.76)	65	0.91 (0.45–1.85)	52	1.12 (0.57–2.17)	323	1.08 (0.80–1.45)	0.99	
*P*-value		0.36		0.99		0.58		0.80		0.75		0.60		
Post current	76	0.85 (0.30–2.43)	64	1.44 (0.75–2.78)	13	1.09 (0.15–7.91)	147	1.45 (0.91–2.32)	46	1.46 (0.70–3.04)	346	1.35 (0.99–1.83)	0.76	
*P*-value		0.77		0.28		0.93		0.12		0.32		0.057		

Abbreviations: BMI=body mass index; CI=confidence interval; HRT=hormone replacement therapy; RR=relative risk; NSAIDs=non-steroidal anti-inflammatory drugs.

a Adjusted for sex (except in models for menopausal status and HRT use), race (except in models for each ethnic group), BMI (except in models for BMI), smoking (except in models for smoking), NSAIDs use (except in models for NSAIDs use), education, alcohol intake, saturated fat intake, unsaturated fat intake, dietary fiber intake, physical activity and family history of colorectal cancer, and stratified on age at baseline questionnaire.

b *P*-value for interaction between ethnic group and diabetes.

c *P*-value for interaction between BMI, age, smoking, NSAIDs use, menopausal status and HRT use and diabetes.

d Pre=premenopausal; Post never=postmenopausal HRT never users; Post past=postmenopausal HRT past users; Post current=postmenopausal HRT current users. Only includes women.
