# Supplementary material for: The association of diabetes with colorectal cancer risk: the Multiethnic Cohort
Source: Br J Cancer. 2010 Jun 8;103(1):120–6. doi: 10.1038/sj.bjc.6605721 (PMC2905298; doi:10.1038/sj.bjc.6605721)
Supplement: Supplementary Tables 1–3 [file 6605721x1.doc]

**Supplemental table1**. Multivariate Relative Risk ^a^ of Colorectal Cancer Associated with Diabetes Status in Men in the Multiethnic Cohort, Los Angeles, CA and Hawaii (n=89478).

|  | **European Americans** | | **African Americans** | | **Native Hawaiians** | | **Japanese Americans** | | **Latinos** | | **All** | |  |  |
| --- | --- | --- | --- | --- | --- | --- | --- | --- | --- | --- | --- | --- | --- | --- |
|  | cases | RR(95% CI) | cases | RR(95% CI) | cases | RR(95% CI) | cases | RR(95% CI) | cases | RR(95% CI) | cases | RR(95% CI) | ^1^P_Int_ | ^2^P_Int_ |
| **All men** | 359 | 0.96(0.69-1.35) | 323 | 1.02(0.77-1.34) | 117 | 0.97(0.60-1.55) | 712 | 1.15(0.95-1.39) | 410 | 1.31(1.04-1.64) | 1921 | 1.12(0.99-1.26) | 0.65 |  |
| *P value* |  | 0.83 |  | 0.91 |  | 0.9 |  | 0.16 |  | 0.023 |  | 0.063 |  |  |
|  |  |  |  |  |  |  |  |  |  |  |  |  |  |  |
| **Colon** | 265 | 1.01(0.69-1.47) | 255 | 1.04(0.76-1.41) | 78 | 0.85(0.47-1.53) | 469 | 1.22(0.97-1.54) | 286 | 1.23(0.93-1.62) | 1353 | 1.12(0.97-1.28) | 0.73 |  |
| *P value* |  | 0.98 |  | 0.82 |  | 0.58 |  | 0.092 |  | 0.15 |  | 0.12 |  |  |
|  |  |  |  |  |  |  |  |  |  |  |  |  |  |  |
| **Right Colon** | 155 | 1.03(0.63-1.69) | 151 | 1.04(0.70-1.55) | 31 | 1.63(0.70-3.83) | 216 | 1.32(0.95-1.85) | 151 | 1.21(0.82-1.77) | 704 | 1.16(0.96-1.41) | 0.96 |  |
| *P value* |  | 0.91 |  | 0.85 |  | 0.26 |  | 0.95 |  | 0.34 |  | 0.12 |  |  |
|  |  |  |  |  |  |  |  |  |  |  |  |  |  |  |
| **Left Colon** | 104 | 1.05(0.59-1.89) | 99 | 1.04(0.63-1.72) | 43 | 0.52(0.21-1.30) | 244 | 1.17(0.84-1.63) | 128 | 1.23(0.81-1.86) | 618 | 1.08(0.87-1.32) | 0.46 |  |
| *P value* |  | 0.86 |  | 0.88 |  | 0.16 |  | 0.36 |  | 0.33 |  | 0.49 |  |  |
|  |  |  |  |  |  |  |  |  |  |  |  |  |  |  |
| **Rectum** | 94 | 0.81(0.39-1.72) | 68 | 0.95(0.50-1.81) | 39 | 1.27(0.57-2.81) | 243 | 1.01(0.71-1.42) | 124 | 1.51(1.00-2.27) | 568 | 1.11(0.89-1.39) | 0.61 |  |
| *P value* |  | 0.59 |  | 0.88 |  | 0.56 |  | 0.98 |  | 0.050 |  | 0.33 |  |  |
|  |  |  |  |  |  |  |  |  |  |  |  |  |  |  |
| **Localized** | 152 | 1.16(0.72-1.88) | 116 | 0.96(0.61-1.54) | 62 | 0.92(0.48-1.77) | 336 | 1.08(0.81-1.43) | 154 | 1.16(0.78-1.72) | 820 | 1.06(0.88-1.27) | 0.82 |  |
| *P value* |  | 0.54 |  | 0.88 |  | 0.81 |  | 0.59 |  | 0.46 |  | 0.55 |  |  |
|  |  |  |  |  |  |  |  |  |  |  |  |  |  |  |
| **Regional** | 180 | 0.75(0.44-1.27) | 154 | 0.93(0.62-1.41) | 50 | 1.05(0.52-2.14) | 333 | 1.19(0.90-1.58) | 194 | 1.51(1.10-2.08) | 911 | 1.15(0.97-1.36) | 0.16 |  |
| *P value* |  | 0.28 |  | 0.74 |  | 0.88 |  | 0.22 |  | 0.011 |  | 0.10 |  |  |
|  |  |  |  |  |  |  |  |  |  |  |  |  |  |  |
| **BMI (kg/m^2^)** |  |  |  |  |  |  |  |  |  |  |  |  |  |  |
| **<25** | 123 | 1.01(0.46-2.19) | 88 | 1.73(0.98-3.05) | 25 | 4.28(1.23-14.88) | 368 | 1.04(0.77-1.40) | 114 | 1.43(0.89-2.28) | 718 | 1.20(0.97-1.49) | 0.49 | 0.43 |
| *P value* |  | 0.98 |  | 0.061 |  | 0.022 |  | 0.79 |  | 0.14 |  | 0.09 |  |  |
| **≥ 25** | 233 | 1.02(0.71-1.48) | 226 | 0.91(0.66-1.26) | 92 | 0.91(0.54-1.53) | 344 | 1.27(0.99-1.64) | 291 | 1.27(0.98-1.66) | 1186 | 1.12(0.98-1.29) | 0.31 |  |
| *P value* |  | 0.90 |  | 0.58 |  | 0.71 |  | 0.062 |  | 0.075 |  | 0.10 |  |  |
|  |  |  |  |  |  |  |  |  |  |  |  |  |  |  |
| **Age** |  |  |  |  |  |  |  |  |  |  |  |  |  |  |
| **<60** | 88 | 0.83(0.37-1.87) | 88 | 0.69(0.37-1.29) | 47 | 1.47(0.73-2.94) | 178 | 0.75(0.47-1.20) | 109 | 0.95(0.57-1.59) | 510 | 0.87(0.67-1.13) | 0.69 | 0.097 |
| *P value* |  | 0.65 |  | 0.24 |  | 0.28 |  | 0.23 |  | 0.85 |  | 0.29 |  |  |
| **60-69** | 157 | 1.12(0.70-1.79) | 128 | 1.16(0.76-1.76) | 53 | 0.98(0.49-1.97) | 303 | 1.35(1.02-1.78) | 223 | 1.49(1.11-2.01) | 864 | 1.30(1.10-1.53) | 0.59 |  |
| *P value* |  | 0.63 |  | 0.50 |  | 0.96 |  | 0.036 |  | 0.008 |  | 0.002 |  |  |
| **≥ 70** | 114 | 0.82(0.44-1.51) | 107 | 1.16(0.72-1.86) | 17 | -- | 231 | 1.13(0.81-1.57) | 78 | 1.29(0.76-2.19) | 547 | 1.04(0.83-1.29) | 0.047 |  |
| *P value* |  | 0.52 |  | 0.55 |  | -- |  | 0.46 |  | 0.35 |  | 0.75 |  |  |
|  |  |  |  |  |  |  |  |  |  |  |  |  |  |  |
| **Smoking** |  |  |  |  |  |  |  |  |  |  |  |  |  |  |
| **Never** | 88 | 0.58(0.25-1.37) | 91 | 1.14(0.66-1.96) | 29 | 2.21(0.85-5.75) | 159 | 1.42(0.95-2.10) | 96 | 1.22(0.74-2.02) | 463 | 1.20(0.95-1.53) | 0.40 | 0.0069 |
| *P value* |  | 0.22 |  | 0.64 |  | 0.11 |  | 0.085 |  | 0.44 |  | 0.13 |  |  |
| **Past** | 202 | 1.23(0.82-1.84) | 165 | 1.12(0.78-1.61) | 56 | 0.63(0.31-1.31) | 419 | 1.24(0.97-1.57) | 243 | 1.28(0.96-1.72) | 1085 | 1.18(1.02-1.37) | 0.41 |  |
| *P value* |  | 0.32 |  | 0.55 |  | 0.22 |  | 0.083 |  | 0.096 |  | 0.029 |  |  |
| **Current** | 67 | 0.55(0.19-1.59) | 66 | 0.50(0.22-1.14) | 31 | 1.40(0.51-3.79) | 129 | 0.47(0.24-0.95) | 65 | 1.32(0.71-2.42) | 358 | 0.74(0.53-1.03) | 0.18 |  |
| *P value* |  | 0.27 |  | 0.10 |  | 0.51 |  | 0.034 |  | 0.38 |  | 0.071 |  |  |
|  |  |  |  |  |  |  |  |  |  |  |  |  |  |  |
| **NSAIDs** |  |  |  |  |  |  |  |  |  |  |  |  |  |  |
| **No** | 193 | 0.95(0.58-1.55) | 152 | 0.90(0.58-1.40) | 72 | 1.18(0.64-2.16) | 479 | 1.31(1.03-1.65) | 218 | 1.40(1.02-1.92) | 1114 | 1.19(1.02-1.39) | 0.47 | 0.53 |
| *P value* |  | 0.83 |  | 0.63 |  | 0.60 |  | 0.027 |  | 0.037 |  | 0.032 |  |  |
| **Yes** | 159 | 1.05(0.65-1.68) | 152 | 1.09(0.74-1.61) | 41 | 0.74(0.33-1.64) | 218 | 0.99(0.70-1.38) | 176 | 1.20(0.84-1.71) | 746 | 1.06(0.88-1.27) | 0.90 |  |
| *P value* |  | 0.85 |  | 0.65 |  | 0.46 |  | 0.93 |  | 0.31 |  | 0.55 |  |  |

^a^ Adjusted for age, race (except in models for each ethnic group), BMI (except in models for BMI), smoking (except in models for smoking), NSAIDs use (except in models for NSAIDs use), education, alcohol intake, saturated fat intake, unsaturated fat intake, dietary fiber intake, physical activity and family history of colorectal cancer, stratified on age at baseline questionnaire.

^1^ Interaction between ethnic group and diabetes.

^2^ Interaction between BMI, age, smoking status, NSAIDs use and diabetes.

**Supplemental table2**. Multivariate Relative Risk ^a^ of Colorectal Cancer Associated with Diabetes Status in Women in the Multiethnic Cohort, Los Angeles, CA and Hawaii (n=109664).

|  | **European Americans** | | **African Americans** | | **Native Hawaiians** | | **Japanese Americans** | | **Latinos** | | **All** | |  |  |
| --- | --- | --- | --- | --- | --- | --- | --- | --- | --- | --- | --- | --- | --- | --- |
|  | cases | RR(95% CI) | cases | RR(95% CI) | cases | RR(95% CI) | cases | RR(95% CI) | cases | RR(95% CI) | cases | RR(95% CI) | ^1^P_Int_ | ^2^P_Int_ |
| **All women** | 305 | 1.43(1.00-2.04) | 468 | 1.26(1.01-1.58) | 88 | 0.82(0.46-1.44) | 485 | 1.49(1.18-1.90) | 282 | 1.09(0.82-1.45) | 1628 | 1.27(1.12-1.45) | 0.32 |  |
| *P value* |  | 0.051 |  | 0.039 |  | 0.48 |  | 0.001 |  | 0.56 |  | <0.001 |  |  |
|  |  |  |  |  |  |  |  |  |  |  |  |  |  |  |
| **Colon** | 236 | 1.47(0.98-2.21) | 387 | 1.39(1.10-1.77) | 66 | 0.83(0.43-1.60) | 378 | 1.53(1.17-2.01) | 208 | 0.93(0.66-1.32) | 1275 | 1.30(1.12-1.50) | 0.19 |  |
| *P value* |  | 0.06 |  | 0.007 |  | 0.58 |  | 0.002 |  | 0.69 |  | <0.001 |  |  |
|  |  |  |  |  |  |  |  |  |  |  |  |  |  |  |
| **Right Colon** | 152 | 1.73(1.07-2.83) | 246 | 1.25(0.92-1.70) | 37 | 0.86(0.36-2.05) | 207 | 0.67(1.17-2.38) | 118 | 0.90(0.57-1.43) | 760 | 1.29(1.07-1.56) | 0.23 |  |
| *P value* |  | 0.027 |  | 0.15 |  | 0.74 |  | 0.004 |  | 0.66 |  | 0.007 |  |  |
|  |  |  |  |  |  |  |  |  |  |  |  |  |  |  |
| **Left Colon** | 78 | 1.19(0.57-2.47) | 124 | 1.74(1.16-2.62) | 28 | 0.85(0.31-2.35) | 162 | 1.44(0.94-2.20) | 81 | 0.92(0.53-1.62) | 473 | 1.34(1.06-1.70) | 0.56 |  |
| *P value* |  | 0.64 |  | 0.008 |  | 0.76 |  | 0.091 |  | 0.78 |  | 0.014 |  |  |
|  |  |  |  |  |  |  |  |  |  |  |  |  |  |  |
| **Rectum** | 69 | 1.32(0.61-2.88) | 81 | 0.73(0.40-1.35) | 22 | 0.74(0.23-2.36) | 107 | 1.36(0.80-2.30) | 74 | 1.61(0.97-2.69) | 353 | 1.19(0.90-1.57) | 0.24 |  |
| *P value* |  | 0.48 |  | 0.31 |  | 0.61 |  | 0.25 |  | 0.067 |  | 0.22 |  |  |
|  |  |  |  |  |  |  |  |  |  |  |  |  |  |  |
| **Localized** | 125 | 1.26(0.71-2.22) | 162 | 1.64(1.14-2.35) | 40 | 0.89(0.39-2.00) | 215 | 1.61(1.14-2.27) | 98 | 1.25(0.78-2.00) | 640 | 1.44(1.18-1.76) | 0.71 |  |
| *P value* |  | 0.43 |  | 0.007 |  | 0.77 |  | 0.007 |  | 0.36 |  | <0.001 |  |  |
|  |  |  |  |  |  |  |  |  |  |  |  |  |  |  |
| **Regional** | 137 | 1.68(1.00-2.80) | 223 | 0.96(0.68-1.36) | 47 | 0.73(0.33-1.61) | 246 | 1.35(0.95-1.91) | 122 | 1.05(0.68-1.62) | 775 | 1.14(0.94-1.37) | 0.33 |  |
| *P value* |  | 0.048 |  | 0.82 |  | 0.43 |  | 0.092 |  | 0.82 |  | 0.19 |  |  |
|  |  |  |  |  |  |  |  |  |  |  |  |  |  |  |
| **BMI (kg/m^2^)** |  |  |  |  |  |  |  |  |  |  |  |  |  |  |
| **<25** | 143 | 0.71(0.26-1.94) | 117 | 1.69(1.00-2.85) | 21 | 0.48(0.054-4.35) | 327 | 1.33(0.94-1.87) | 70 | 1.15(0.56-2.37) | 678 | 1.27(0.99-1.64) | 0.47 | 0.08 |
| *P value* |  | 0.5 |  | 0.049 |  | 0.52 |  | 0.11 |  | 0.7 |  | 0.061 |  |  |
| **≥ 25** | 156 | 1.76(1.19-2.61) | 331 | 1.18(0.92-1.52) | 64 | 0.89(0.49-1.62) | 149 | 1.78(1.26-2.52) | 202 | 1.08(0.79-1.49) | 902 | 1.31(1.12-1.52) | 0.09 |  |
| *P value* |  | 0.005 |  | 0.18 |  | 0.71 |  | 0.001 |  | 0.63 |  | 0.001 |  |  |
|  |  |  |  |  |  |  |  |  |  |  |  |  |  |  |
| **Age** |  |  |  |  |  |  |  |  |  |  |  |  |  |  |
| **<60** | 79 | 1.78(0.81-3.90) | 139 | 1.07(0.69-1.66) | 42 | 0.84(0.37-1.91) | 112 | 2.42(1.49-3.93) | 90 | 1.04(0.61-1.79) | 462 | 1.32(1.03-1.70) | 0.030 | 0.12 |
| *P value* |  | 0.15 |  | 0.75 |  | 0.68 |  | <0.001 |  | 0.88 |  | 0.027 |  |  |
| **60-69** | 124 | 1.73(1.06-2.83) | 171 | 1.44(1.01-2.06) | 36 | 0.53(0.20-1.43) | 204 | 1.50(1.05-2.15) | 140 | 1.17(0.79-1.73) | 675 | 1.36(1.12-1.64) | 0.16 |  |
| *P value* |  | 0.028 |  | 0.044 |  | 0.21 |  | 0.027 |  | 0.43 |  | 0.002 |  |  |
| **≥ 70** | 102 | 0.83(0.39-1.75) | 158 | 1.22(0.84-1.79) | 10 | -- | 169 | 1.00(0.64-1.56) | 52 | 1.00(0.51-1.94) | 491 | 1.07(0.84-1.36) | 0.64 |  |
| *P value* |  | 0.62 |  | 0.30 |  | -- |  | 0.99 |  | 1.00 |  | 0.59 |  |  |
|  |  |  |  |  |  |  |  |  |  |  |  |  |  |  |
| **Smoking** |  |  |  |  |  |  |  |  |  |  |  |  |  |  |
| **Never** | 123 | 1.52(0.87-2.66) | 196 | 1.46(1.04-2.04) | 31 | 0.71(0.26-1.92) | 325 | 1.58(1.18-2.12) | 163 | 1.22(0.84-1.76) | 838 | 1.38(1.16-1.64) | 0.65 | 0.51 |
| *P value* |  | 0.14 |  | 0.028 |  | 0.50 |  | 0.002 |  | 0.30 |  | <0.001 |  |  |
| **Past** | 123 | 1.61(0.96-2.72) | 182 | 0.97(0.67-1.40) | 43 | 0.93(0.43-2.00) | 109 | 1.60(0.99-2.59) | 84 | 1.05(0.62-1.77) | 541 | 1.20(0.96-1.49) | 0.30 |  |
| *P value* |  | 0.071 |  | 0.87 |  | 0.85 |  | 0.057 |  | 0.87 |  | 0.11 |  |  |
| **Current** | 54 | 0.89(0.26-3.01) | 85 | 1.85(1.08-3.15) | 12 | 2.37(0.14-41.17) | 46 | 0.63(0.23-1.73) | 28 | 0.98(0.33-2.93) | 225 | 1.17(0.80-1.72) | 0.59 |  |
| *P value* |  | 0.85 |  | 0.024 |  | 0.56 |  | 0.37 |  | 0.97 |  | 0.41 |  |  |
|  |  |  |  |  |  |  |  |  |  |  |  |  |  |  |
| **NSAIDs** |  |  |  |  |  |  |  |  |  |  |  |  |  |  |
| **No** | 156 | 1.19(0.69-2.05) | 235 | 1.17(0.84-1.62) | 51 | 0.53(0.22-1.27) | 358 | 1.45(1.08-1.95) | 150 | 0.92(0.61-1.39) | 950 | 1.16(0.97-1.39) | 0.26 | 0.25 |
| *P value* |  | 0.53 |  | 0.36 |  | 0.15 |  | 0.012 |  | 0.68 |  | 0.096 |  |  |
| **Yes** | 132 | 1.51(0.89-2.56) | 200 | 1.41(1.02-1.95) | 34 | 1.22(0.52-2.85) | 106 | 1.38(0.85-2.23) | 107 | 1.36(0.87-2.13) | 579 | 1.39(1.13-1.70) | 0.99 |  |
| *P value* |  | 0.13 |  | 0.040 |  | 0.64 |  | 0.19 |  | 0.17 |  | 0.001 |  |  |
|  |  |  |  |  |  |  |  |  |  |  |  |  |  |  |
| **Menopausal status and HRT use ^b^** | | |  |  |  |  |  |  |  |  |  |  |  |  |
| **Pre** | 12 | 8.74(1.30-58.70) | 21 | 0.47(0.088-2.55) | 13 | 0.79(0.15-4.26) | 29 | 4.83(1.95-11.95) | 13 | 2.83(0.40-19.91) | 88 | 2.04(1.17-3.57) | 0.040 | 0.080 |
| *P value* |  | 0.026 |  | 0.39 |  | 0.79 |  | 0.001 |  | 0.30 |  | 0.012 |  |  |
| **Post never** | 124 | 1.34(0.79-2.27) | 209 | 1.60(1.17-2.19) | 40 | 0.57(0.24-1.37) | 212 | 1.42(0.99-2.03) | 136 | 1.01(0.67-1.52) | 721 | 1.30(1.08-1.57) | 0.26 |  |
| *P value* |  | 0.28 |  | 0.003 |  | 0.21 |  | 0.059 |  | 0.96 |  | 0.005 |  |  |
| **Post past** | 74 | 1.42(0.67-2.97) | 116 | 1.00(0.62-1.62) | 16 | 1.65(0.28-9.76) | 65 | 0.91(0.45-1.85) | 52 | 1.12(0.57-2.17) | 323 | 1.08(0.80-1.45) | 0.99 |  |
| *P value* |  | 0.36 |  | 0.99 |  | 0.58 |  | 0.80 |  | 0.75 |  | 0.60 |  |  |
| **Post current** | 76 | 0.85(0.30-2.43) | 64 | 1.44(0.75-2.78) | 13 | 1.09(0.15-7.91) | 147 | 1.45(0.91-2.32) | 46 | 1.46(0.70-3.04) | 346 | 1.35(0.99-1.83) | 0.76 |  |
| *P value* |  | 0.77 |  | 0.28 |  | 0.93 |  | 0.12 |  | 0.32 |  | 0.057 |  |  |

^a^ Adjusted for age, race (except in models for each ethnic group), BMI (except in models for BMI), smoking (except in models for smoking), NSAIDs use (except in models for NSAIDs use), menopausal status and HRT use (except in models for menopausal status and HRT use), education, alcohol intake, saturated fat intake, unsaturated fat intake, dietary fiber intake, physical activity and family history of colorectal cancer, stratified on age at baseline questionnaire.

^b^ Pre: premenopausal women; Post never: postmenopausal HRT never users; Post past: postmenopausal HRT past users; Post current: postmenopausal HRT current users.

^1^ P value for interaction between ethnic group and diabetes.

^2^ P value for interaction between BMI, age, smoking status, NSAIDs use, menopausal status & HRT use and diabetes.

**Supplemental table 3.**  Multivariate Relative Risks^a^ of Colorectal Cancer Associated with Diabetes Status According to Site and Stage of Disease, and Smoking Status (never, past and current) in the Multiethnic Cohort, Los Angeles, CA and Hawaii

|  | **European Americans** | | **African Americans** | | **Native Hawaiians** | | **Japanese Americans** | | **Latinos** | | **All** | |  |  |
| --- | --- | --- | --- | --- | --- | --- | --- | --- | --- | --- | --- | --- | --- | --- |
|  | cases | RR(95% CI) | cases | RR(95% CI) | cases | RR(95% CI) | cases | RR(95% CI) | cases | RR(95% CI) | cases | RR(95% CI) | ^1^P_Int_ | ^2^P_Int_ |
| **Colon** |  |  |  |  |  |  |  |  |  |  |  |  |  |  |
| **never** | 171 | 0.92(0.54-1.58) | 247 | 1.42(1.05-1.92)) | 46 | 0.94(0.44-2.02) | 359 | 1.51(1.15-1.99) | 183 | 1.04(0.72-1.51) | 1006 | 1.26(1.07-1.49) | 0.37 | 0.18 |
| *P value* |  | 0.77 |  | 0.021 |  | 0.88 |  | 0.003 |  | 0.82 |  | 0.005 |  |  |
| **past** | 242 | 1.47(1.03-2.09) | 277 | 1.12(0.85-1.49) | 64 | 0.84(0.45-1.57) | 373 | 1.45(1.14-1.86) | 238 | 1.02(0.75-1.40) | 1194 | 1.22(1.06-1.40) | 0.12 |  |
| *P value* |  | 0.035 |  | 0.41 |  | 0.59 |  | 0.003 |  | 0.89 |  | 0.006 |  |  |
| **current** | 81 | 0.84(0.35-2.00) | 112 | 1.24(0.76-2.02) | 31 | 0.78(0.25-2.39) | 106 | 0.49(0.23-1.02) | 63 | 1.62(0.90-2.92) | 393 | 0.99(0.74-1.32) | 0.16 |  |
| *P value* |  | 0.70 |  | 0.40 |  | 0.67 |  | 0.057 |  | 0.11 |  | 0.94 |  |  |
| **Right colon** |  |  |  |  |  |  |  |  |  |  |  |  |  |  |
| **never** | 104 | 0.96(0.49-1.90) | 160 | 1.16(0.78-1.72) | 21 | 1.48(0.51-4.34) | 205 | 1.71(1.21-2.42) | 105 | 0.90(0.54-1.48) | 595 | 1.22(0.99-1.51) | 0.25 | 0.80 |
| *P value* |  | 0.92 |  | 0.47 |  | 0.47 |  | 0.002 |  | 0.68 |  | 0.065 |  |  |
| **past** | 143 | 1.53(0.97-2.43) | 167 | 1.26(0.88-1.79) | 25 | 1.50(0.59-3.78) | 161 | 1.42(0.97-2.07) | 123 | 0.89(0.57-1.40) | 619 | 1.25(1.02-1.52) | 0.22 |  |
| *P value* |  | 0.069 |  | 0.21 |  | 0.39 |  | 0.069 |  | 0.62 |  | 0.029 |  |  |
| **current** | 57 | 1.18(0.45-3.13) | 67 | 1.01(0.52-1.95) | 20 | 0.67(0.13-3.32) | 51 | 0.78(0.33-1.89) | 35 | 2.29(1.10-4.80) | 230 | 1.19(0.83-1.71) | 0.2 |  |
| *P value* |  | 0.74 |  | 0.98 |  | 0.62 |  | 0.59 |  | 0.027 |  | 0.35 |  |  |
| **Left colon** |  |  |  |  |  |  |  |  |  |  |  |  |  |  |
| **never** | 64 | 0.90(0.37-2.17) | 80 | 1.91(1.16-3.15) | 23 | 0.50(0.14-1.78) | 147 | 1.21(0.76-1.93) | 73 | 1.18(0.66-2.11) | 387 | 1.26(0.97-1.65) | 0.39 | 0.11 |
| *P value* |  | 0.82 |  | 0.012 |  | 0.29 |  | 0.43 |  | 0.58 |  | 0.086 |  |  |
| **past** | 91 | 1.55(0.88-2.74) | 99 | 1.05(0.66-1.69) | 38 | 0.57(0.23-1.42) | 203 | 1.60(1.15-2.22) | 107 | 1.18(0.76-1.83) | 538 | 1.26(1.03-1.56) | 0.14 |  |
| *P value* |  | 0.13 |  | 0.83 |  | 0.23 |  | 0.006 |  | 0.47 |  | 0.027 |  |  |
| **current** | 23 | 0.38(0.048-2.95) | 41 | 1.65(0.75-3.65) | 9 | 1.82(0.21-16.08) | 53 | 0.24(0.057-1.00) | 27 | 0.73(0.24-2.27) | 153 | 0.74(0.45-1.23) | 0.21 |  |
| *P value* |  | 0.35 |  | 0.21 |  | 0.59 |  | 0.051 |  | 0.59 |  | 0.25 |  |  |
| **Rectum** |  |  |  |  |  |  |  |  |  |  |  |  |  |  |
| **never** | 40 | 1.60(0.64-4.00) | 40 | 1.00(0.43-2.32) | 14 | 1.73(0.45-6.63) | 125 | 1.62(1.03-2.54) | 76 | 1.85(1.11-3.07) | 295 | 1.54(1.16-2.04) | 0.58 | 0.0019 |
| *P value* |  | 0.18 |  | 0.99 |  | 0.43 |  | 0.036 |  | 0.017 |  | 0.003 |  |  |
| **past** | 83 | 1.08(0.52-2.21) | 70 | 0.75(0.39-1.42) | 35 | 0.62(0.25-1.56) | 155 | 0.97(0.64-1.48) | 89 | 1.79(1.14-2.83) | 432 | 1.10(0.86-1.40) | 0.12 |  |
| *P value* |  | 0.84 |  | 0.37 |  | 0.31 |  | 0.90 |  | 0.012 |  | 0.47 |  |  |
| **current** | 40 | 0.31(0.04-2.35) | 39 | 0.90(0.36-2.24) | 12 | -- | 69 | 0.63(0.27-1.49) | 30 | 0.49(0.14-1.67) | 190 | 0.71(0.44-1.15) | 0.5 |  |
| *P value* |  | 0.26 |  | 0.82 |  | -- |  | 0.29 |  | 0.25 |  | 0.17 |  |  |
| **Localized** |  |  |  |  |  |  |  |  |  |  |  |  |  |  |
| **never** | 81 | 0.99(0.46-2.12) | 97 | 1.84(1.16-2.92) | 27 | 0.87(0.34-2.25) | 214 | 1.51(1.06-2.15) | 85 | 1.30(0.77-2.20) | 504 | 1.41(1.13-1.77) | 0.79 | 0.30 |
| *P value* |  | 0.97 |  | 0.01 |  | 0.77 |  | 0.023 |  | 0.32 |  | 0.003 |  |  |
| **past** | 149 | 1.37(0.86-2.16) | 131 | 1.06(0.70-1.61) | 54 | 0.71(0.35-1.46) | 257 | 1.33(0.98-1.79) | 124 | 1.17(0.77-1.79) | 715 | 1.16(0.97-1.40) | 0.22 |  |
| *P value* |  | 0.18 |  | 0.78 |  | 0.36 |  | 0.065 |  | 0.46 |  | 0.11 |  |  |
| **current** | 46 | 1.13(0.39-3.31) | 50 | 1.17(0.56-2.45) | 20 | 4.28(1.22-15.03) | 74 | 0.61(0.27-1.36) | 39 | 1.26(0.58-2.74) | 229 | 1.06(0.73-1.54) | 0.62 |  |
| *P value* |  | 0.82 |  | 0.67 |  | 0.023 |  | 0.23 |  | 0.56 |  | 0.75 |  |  |
| **Regional** |  |  |  |  |  |  |  |  |  |  |  |  |  |  |
| **never** | 107 | 0.98(0.51-1.86) | 139 | 0.97(0.63-1.51) | 31 | 1.38(0.55-3.44) | 244 | 1.43(1.02-1.99) | 121 | 1.29(0.84-1.98) | 642 | 1.21(0.98-1.48) | 0.63 | 0.037 |
| *P value* |  | 0.94 |  | 0.90 |  | 0.50 |  | 0.037 |  | 0.25 |  | 0.075 |  |  |
| **past** | 150 | 1.26(0.77-2.06) | 164 | 1.00(0.69-1.46) | 44 | 0.83(0.39-1.78) | 241 | 1.34(0.97-1.83) | 155 | 1.26(0.88-1.81) | 754 | 1.20(1.00-1.44) | 0.75 |  |
| *P value* |  | 0.35 |  | 1.00 |  | 0.64 |  | 0.074 |  | 0.21 |  | 0.045 |  |  |
| **current** | 57 | 0.57(0.17-1.91) | 69 | 0.96(0.48-1.94) | 20 | 0.52(0.11-2.43) | 90 | 0.43(0.18-0.99) | 34 | 1.90(0.86-4.16) | 270 | 0.79(0.54-1.16) | 0.18 |  |
| *P value* |  | 0.36 |  | 0.92 |  | 0.41 |  | 0.049 |  | 0.11 |  | 0.23 |  |  |

^a^Adjusted for age, sex, race (except in models for each ethnic group), BMI, education, alcohol intake, NSAIDs use, saturated fat intake, unsaturated fat intake, dietary fiber intake, physical activity and family history of colorectal cancer.

^1^ P value for interactions between ethnic groups and diabetes.

^2^ P value for interactions between smoking status and diabetes.
